# Meetings of Societies

**Published:** 1924-07

**Authors:** 


### MEETINGS OF SOCIETIES.

### Bristol Medico-Chirurgical Society.

*April 9th*, 1924.

Mr. J. Lacy Firth, President, in the Chair.

Mr. W. A. Adams showed a case of "**Endothoracic Goitre**" (this case will be reported in our next issue), and read notes on a case of Vesico-Colic Fistula.

Mr. Arthur Mitchell (visitor) demonstrated an apparatus for transmitting the sounds of the heart to an ordinary wireless "loud speaker." He stated that three years ago he and Dr. Alan Fawcett had experimented in this direction, that he had now succeeded in making an improved microphone which, though far from perfect, was a great improvement on the original one, and then demonstrated the normal heart sounds to the meeting. He stated he hoped in time to perfect this microphone, when he would like to demonstrate to the Society again. The patients were in a room adjoining the theatre in which the meeting was held. The character of the heart sounds was quite clearly heard.

Dr. Coombs and Dr. Herapath read a paper on "**Auricular Failure.**" (This paper will be published in our next issue.)

---

*May 14th*, 1924.

Mr. J. Lacy Firth, President, in the Chair.

Dr. Hadfield showed a number of **Pathological Specimens.**

Mr. Eric Watson-Williams showed five cases with symptoms of disease of the internal ear. Certain of the phenomena of these conditions were demonstrated, especially the alterations of gait. The notes on these cases are embodied in the article on "**Labyrinthitis**" (*vide* p. 135).

Mr. C. F. Walters read a paper on "**A Few Suggestions in General Surgery**" (illustrated by the cinematograph).

